# Manufacturing of LDPE-Based Shields and Exposure in LEO Environment in the MISSE9 Campaign

**DOI:** 10.3390/polym18131634

**Published:** 2026-07-01

**Authors:** Denise Bellisario, Alice Proietti, Leandro Iorio, Fabrizio Quadrini, Loredana Santo

**Affiliations:** 1 Department of Industrial Engineering, University of Rome “Tor Vergata”, via del Politecnico 1, 00133 Rome, Italy; 2 Space Sustainability Center, University of Rome “Tor Vergata”, via del Politecnico 1, 00133 Rome, Italy; 3 Department of Engineering and Science, University of Mercatorum, Piazza Mattei, 10, 00186 Rome, Italy

**Keywords:** shielding, LDPE, space, compression molding, LEO, MISSE, galactic cosmic rays, boron nitride, samarium-cobalt, fillers

## Abstract

During the 9th NASA MISSE (Materials International Space Station Experiment) campaign, a multilayer LDPE-based shield was tested in a low Earth orbit (LEO) environment aboard the International Space Station for the first time, in the wake-facing orientation. The architecture of the multilayer flight sample, 1 inch in diameter, consisted of two external LDPE sheets and two inner layers filled with boron nitride and samarium–cobalt powders. The inner layers were manufactured using an original process based on compression molding of two superimposed LDPE sheets, with the functional filler deposited onto one of them by spray coating. Thanks to the partial filling of the inner layers and their relative positioning, four different shielding configurations were obtained. The sample was exposed to the space environment for approximately 200 days, experiencing the combined effects of vacuum, solar radiation, thermal cycling, and limited atomic oxygen exposure. The results show that the structural integrity of the shield was not affected by its prolonged residence in LEO. The most significant effect observed was the partial oxidation of the external surfaces of the individual layers, particularly the uppermost layer.

## 1. Introduction

Material selection for space exploration is strongly influenced by the adverse effects that high-energy particles in the space radiation environment can have on both humans and electronic devices and infrastructures [1]. Galactic cosmic rays (GCRs), which consist of protons (87%), helium ions (12%), and heavy ions (1%), can penetrate spacecraft shells and affect astronauts through their radiobiological effects [2]. In low Earth orbit (LEO), the contribution of heavy ions to the radiation dose is limited; however, secondary emissions, including low-energy neutrons, have a significant impact on superficial human tissues such as the eyes and skin. Deep tissues, such as bones, are instead severely affected by both residual radiation and fast neutrons [3]. Since radiation represents a hazard to the human central nervous system [4], radiation shielding plays a crucial role not only in space applications but also in terrestrial environments. Indeed, workers in nuclear power plants, as well as in certain healthcare and aerospace industries, may be exposed to radiation [5,6].

The operational lifetime of electronic devices used in LEO is also limited by radiation-induced damage to the semiconductor components contained within them [7]. These considerations highlight the importance of GCR shielding in the design and planning of long-duration deep-space missions [8]. GCR shield design can be based either on radiation deflection or radiation absorption. In the former case, radiation is deflected by the action of a magnetic field, and the shield is referred to as “active.” In the latter case, radiation is absorbed and converted into heat, and the shield is defined as “passive” [9].

In the passive approach, shielding against protons and heavy ions can be achieved using low-atomic-number elements and hydrogen-rich materials. Indeed, when high-energy particles interact with the atoms of conventional spacecraft materials, fragmentation processes may occur, generating secondary radiation [10,11]. The shielding effectiveness of hydrogen-rich polyethylene (PE) has already been evaluated aboard the International Space Station (ISS) [12,13]. Furthermore, a 100 mm thick PE layer combined with an active shield was found to reduce the absorbed dose rate by 1.9%, 7.3%, and 27.9% for protons, alpha particles, and carbon ions, respectively [14]. Other thermoplastics have also been investigated, including polyoxymethylene [15] and polyether ether ketone (PEEK)-based composites [2], which achieved a shielding effectiveness up to 17% greater than that of aluminum (Al) [10].

Low-atomic-mass elements such as boron (B) and nitrogen (N) can also reduce secondary radiation; however, they cannot be used as pure elements for structural applications [9]. For this reason, compounds such as boron nitride (BN) have been investigated. Indeed, thermoplastic polymers can be readily filled with BN. For example, a polyurethane (PU)-based composite containing 20 wt% BN was found to reduce neutron transmission [16]. Moreover, BN/PU/ultra-high-molecular-weight polyethylene (UHMWPE) composites have been manufactured, showing a reduction in the neutron transmission factor of 0.28% at a BN content of 20 wt% [6]. The beneficial effect of BN addition to high-density polyethylene (HDPE) has also been demonstrated [5,17], as has the effectiveness of incorporating UHMWPE fibers into a hydrogen-rich polybenzoxazine matrix [18,19] or into polyethylene itself [20].

According to the current state of the art, aluminum (Al) and polyethylene (PE) are among the most widely used materials for radiation shielding [10,17]. In particular, the synergistic effect of these materials can reduce the muon flux, thereby improving shielding efficiency [1]. Depending on the orbital environment, replacing Al with PE may provide advantages in terms of both shielding effectiveness and mass reduction [21]. Multifunctional PE-based composites can be obtained through the incorporation of small amounts (1–5 wt%) of nanoreinforcements, such as single-walled carbon nanotubes and graphene oxide nanoplatelets [22]. In addition, the shielding performance of fibrous materials [11] and fiber-reinforced epoxy composites has been investigated [23]. Recent studies have shown that low-density hydrides [24] are particularly promising shielding materials. For example, Al/lithium hydride composites have demonstrated good shielding performance [25,26]. Furthermore, Martian regolith has been shown to provide effective radiation shielding [27,28].

In this work, a PE-based passive shield was manufactured and exposed to the LEO environment using the Materials International Space Station Experiment (MISSE) platform. The sample was installed on the MISSE Flight Facility (MISSE-FF), a permanent external platform of the International Space Station (ISS), in the wake-facing orientation (opposite to the direction of travel). In this configuration, exposure to atomic oxygen and direct solar radiation is reduced. Several facilities have been developed to expose materials to the space environment. One of the most prominent examples is MISSE, a NASA program dedicated to in-space materials testing [29]. Prior to the present experiment, eight MISSE campaigns had already been completed. For the ninth campaign, a new fully automated MISSE-FF platform was introduced. During the first seven campaigns, more than 540 samples were tested in 39 individual flight experiments. A similar facility was also developed by ESA, although its underlying concept differed somewhat, being oriented more toward scientific investigations than technical evaluations. The exposed materials included thermal-control paints and foils, optical glasses, solar-sail materials, and anodized metallic surfaces [30].

**Figure 1:**
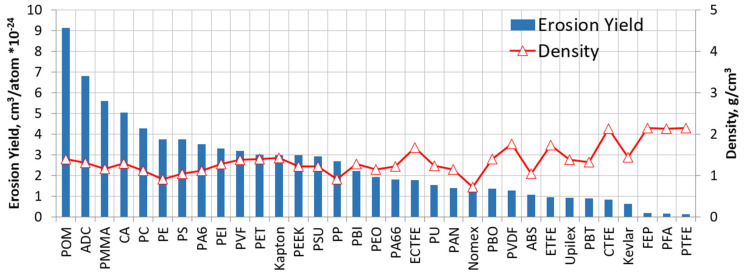
Erosion yield and density of plastic materials exposed to space environment during the MISSE 2 campaign, [28].

Results from the MISSE-2 campaign are shown in Figure 1. Numerous engineering plastics were exposed to the space environment to identify the most suitable candidates for various applications. In that study, mass loss was attributed to atomic oxygen erosion and was measured after 17 months of exposure. The erosion yield was calculated using Kapton as the reference material and was expressed as the volume loss per incident oxygen atom. As shown in Figure 1, fluorinated polymers exhibited the best resistance to atomic oxygen, whereas polyoxymethylene (POM) showed the poorest performance. Polymer density is also reported for comparison and was found to be relatively similar among all materials despite their markedly different erosion behaviors.

**Figure 2:**
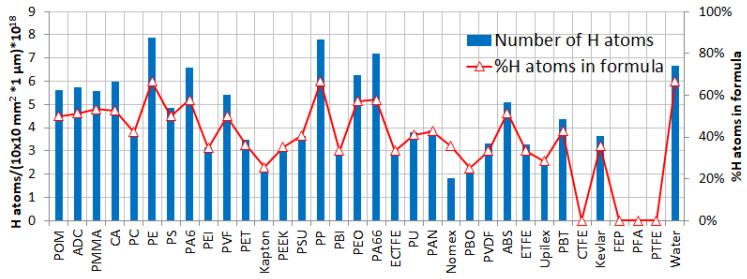
Hydrogen (H) content in plastics exposed during the MISSE2 campaign in terms of amount of atoms in a small thin sheet and percentage of H atoms in the chemical formula.

Unfortunately, the polymers that provide the highest resistance to atomic oxygen are not necessarily the most suitable candidates for GCR shielding, and a compromise must therefore be found for LEO applications. Figure 2 shows the hydrogen content per unit volume, estimated from the chemical formula and density of each polymer. The results are presented both as the number of hydrogen atoms contained in a thin sheet of material measuring 10 × 10 mm^2^ and 1 μm in thickness, and as the percentage of hydrogen atoms relative to the total number of atoms in the polymer chemical formula. Materials with excellent resistance to atomic oxygen, such as FEP, PFA, and PTFE, contain no hydrogen, whereas the highest hydrogen contents are found in polyolefins such as polyethylene (PE) and polypropylene (PP), in which hydrogen accounts for approximately 90% of the total number of atoms.

The adoption of polyethylene (PE) for GCR shielding appears to represent the best compromise currently available and is consistent with prevailing research trends, although the optimal filler system is still under investigation in terms of both shielding performance and manufacturing feasibility. The present study primarily addresses the latter aspect. Specifically, the objective of this work is not to demonstrate the shielding effectiveness of filled PE sheets, which have already been extensively investigated in the literature. Rather, a novel manufacturing route is proposed and validated in the space environment. The approach is based on the compression molding of PE sheets, eliminating the need for a preliminary compounding stage. Indeed, highly filled PE compounds are difficult to process into thin films and are currently more suitable for bulk components. An original multilayer configuration was therefore designed, consisting of a specific stacking sequence of filled and unfilled PE sheets. The inner layers were filled with the well-established boron nitride (BN) and, for the first time, a samarium–cobalt alloy selected for its unique magnetic properties. The two fillers differ significantly in density, which complicates their incorporation into conventional mixed-filler systems. The designed multilayer architecture, combined with the innovative manufacturing route, successfully overcomes this limitation.

## 2. Materials and Methods

### 2.1. Sample Design and Manufacturing

The samples were designed as multilayer polymeric systems with a circular geometry and a nominal diameter of 25.4 mm (1 inch). The overall structure consisted of a stacked configuration of low-density polyethylene (LDPE) sheets, combining both passive and functionalized layers. The outermost layers were composed of neat LDPE sheets acting as passive barriers, 100 µm thick. Inner layers were selectively functionalized to introduce shielding functionalities.

A first layer was obtained by filling LDPE substrates with magnetic particles, such as samarium–cobalt (Sm_2_Co_7_, Sigma-Aldrich, Darmstadt, Germany). This selection was primarily made to facilitate processing of powders that are difficult to incorporate into flexible films. Moreover, there was the idea of locally generating a magnetic field around the particles. Although the intensity of the generated field is not sufficient to deflect high-energy solar or galactic cosmic radiation, its potential influence on radiation–matter interaction mechanisms within the material matrix was considered. Even if this was not a fully active layer, the idea was to partially introduce a degree of activity into the otherwise passive shield.

**Figure 3:**
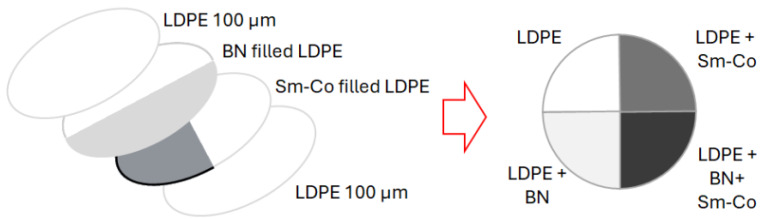
Architecture of the LDPE-based shield for the exposure experiment in MISSE9.

To mitigate possible secondary radiation effects associated with high atomic mass elements such as samarium, an additional passive barrier layer was introduced. This layer consisted of LDPE filled with BN particles (Sigma-Aldrich), selected for their radiation-shielding properties. Inner layers were filled only on one half, while the other half remained unfilled. The architecture of the final sample is shown in Figure 3. The two inner layers were rotated to achieve partial overlap over a 90° circular sector. After assembly of the four sheets, one quarter of the sample consisted of unfilled LDPE, two quarters contained a filled layer (BN or Sm–Co), and the last quarter contained both filled layers. This configuration was adopted to test different shielding compositions simultaneously.

Both the “semi-active” (SmCo-filled) and passive (BN-filled) layers were fabricated in the laboratory using an original procedure, which started with spraying a dispersion of the filler onto thin LDPE films, 60 µm thick. Specifically, hexagonal boron nitride (h-BN) powder (98% purity, average particle size 1 µm) was used as a neutron-absorbing filler. Samarium–cobalt (Sm–Co) powder (CAS No. 12305-84-9, particle size 1–50 µm) was employed as the magnetic functional phase. The materials were used as supplied by the manufacturer.

**Figure 4:**
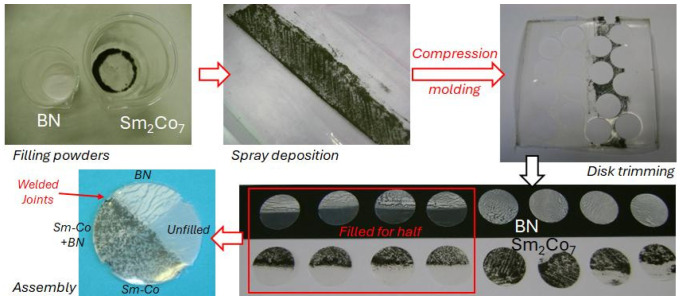
Some stages of the sample manufacturing.

The dispersion was prepared by diluting the fillers in isopropanol. After solvent evaporation, a second LDPE sheet was placed on top of the coated one, and the assembly was compression-molded in a hot parallel-plate press. Figure 4 shows several steps of the manufacturing process. Since the LDPE substrate was partially coated with the two powders, disks were cut so that the fillers were present on only one half.

After stacking, the different layers were joined by partially melting their periphery at four points using a soldering iron.

### 2.2. Exposure in Space Environment

**Figure 5:**
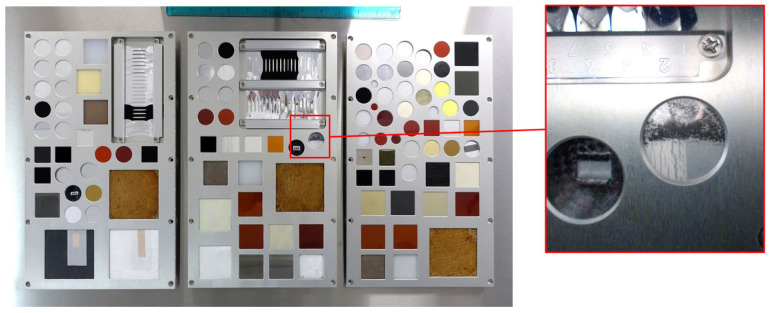
The flight sample M9W-C9 in the carrier of the MISSE-FF platform.

A flight sample was sent to NASA for integration into the carrier of the MISSE-FF platform, together with a backup sample, which will be used as a control at the end of the mission. The sample was named M9W-C9, where M9 refers to the MISSE-9 campaign, W to the wake orientation of the carrier, and C9 to the relative position within the carrier. Figure 5 shows the flight sample inside the carrier, near another sample (M9W-C10F), which is a shape-memory polymer composite.

The MISSE-9 campaign started with the launch of the full MISSE-FF platform aboard SpaceX CRS-14 on 2 April 2018. The carriers were deployed in open space on 19 April 2018, retrieved on 26 April 2019, and returned to Earth on 3 June 2019. Due to technical issues after reintegration into the MISSE-FF platform, the experiments remained inside the closed device for an extended period. Ultimately, the samples were exposed to the space vacuum for 1.07 years and to the LEO environment for 0.54 years. After retrieval, the samples underwent several characterization and analysis procedures, which are still ongoing.

### 2.3. Sample Analysis

After recovery, the flight and backup samples were weighed and disassembled for analysis. This study reports the findings of the first characterization campaign, in which all planned tests are minimally invasive. A first macroscopic evaluation of the sample layers after separation was performed using stereoscopic optical microscopy. These initial images were acquired to provide an overview of the surface condition and to identify visible degradation features induced by the space environment.

This preliminary analysis enabled the assessment of large-scale defects such as discoloration, yellowing, surface irregularities, and cracking by comparing the space-exposed samples with their backup counterparts stored on Earth. Observations were carried out on each circular sample by examining the four quadrants to capture possible spatial heterogeneity. Both the external polymeric layer, directly exposed to the space environment, and the internal layer were analyzed to identify potential gradients in macroscopic degradation.

Measurements were conducted using a Leica S9i stereomicroscope (Leica Camera AG, Wetzlar, Germany). Images were acquired under ambient conditions using reflected-light illumination, ensuring consistent observation parameters across all specimens.

Fourier Transform Infrared (FTIR) spectroscopy was used to investigate the chemical structure and possible degradation phenomena of filled and unfilled polyethylene (LDPE) films exposed to the space environment, in comparison with backup samples stored on Earth and a virgin LDPE reference. The analysis aimed to identify characteristic functional groups of polyethylene and detect potential chemical modifications induced by environmental exposure, such as oxidation or chain scission. FTIR measurements were carried out on different regions of each circular specimen, specifically analyzing the four quadrants to account for possible spatial heterogeneity across the samples. Spectra were collected for both the external polymeric layer—directly exposed to the space environment—and the internal layer, as well as for the corresponding backup samples.

All analyses were performed using a Jasco FT/IR-4X spectrometer (Jasco Corporation, Milan, Italy) equipped with a diamond attenuated total reflectance (ATR) accessory, enabling single or multiple reflections, and a DLaTGS (deuterated L-alanine doped triglycine sulfate) detector.

Measurements were conducted at room temperature over the 6000–400 cm^−1^ spectral range, with a resolution of 4 cm^−1^ and an average of 150 scans per spectrum. Spectral acquisition and processing were performed using Spectra Manager™ Spectroscopy Software version 2.8 (Jasco Corporation, Piazza Cavour, Milan, Italy).

## 3. Results

**Figure 6:**
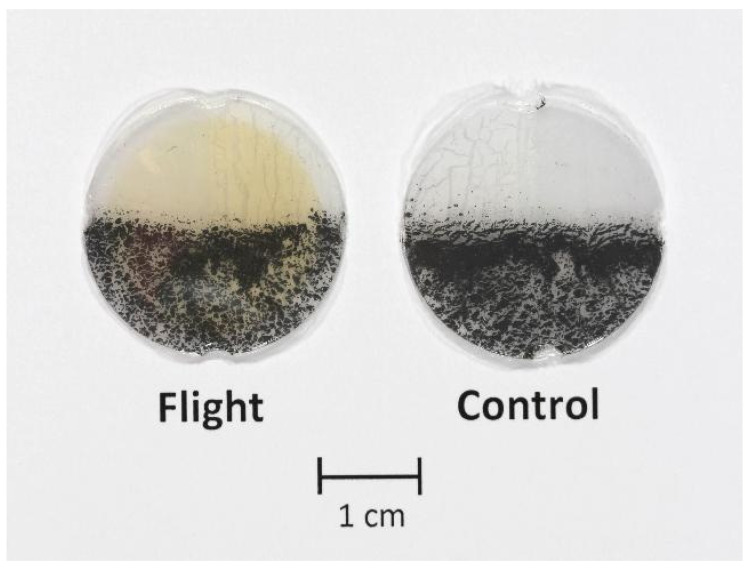
Comparison between the flight and the backup sample in the end of the exposure.

**Figure 7:**
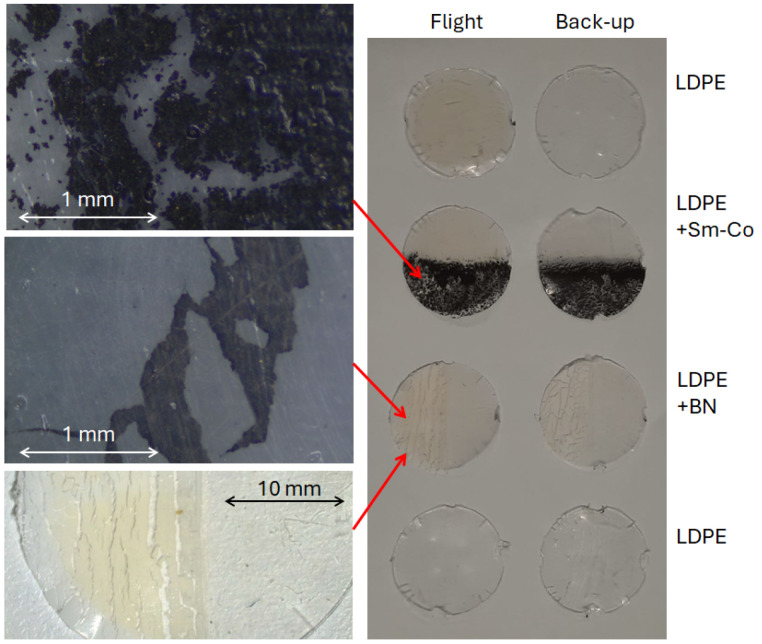
Microscopic observations of the disassembled layers of the samples.

Table 1 summarizes the physical properties of the flight and backup samples before and after exposure. Only the flight sample was subjected to environmental aging in space, while the backup sample was stored under controlled conditions at room temperature, standard humidity, and without any irradiation. The appearance of the flight sample after exposure is shown in Figure 6, together with the backup sample used as a control. The main results of the microscopic analysis are reported in Figure 7. Scanning electron microscopy was not performed at this stage to avoid potential surface contamination, as it requires metal coating of the sample surface for analysis.

**Figure 8:**
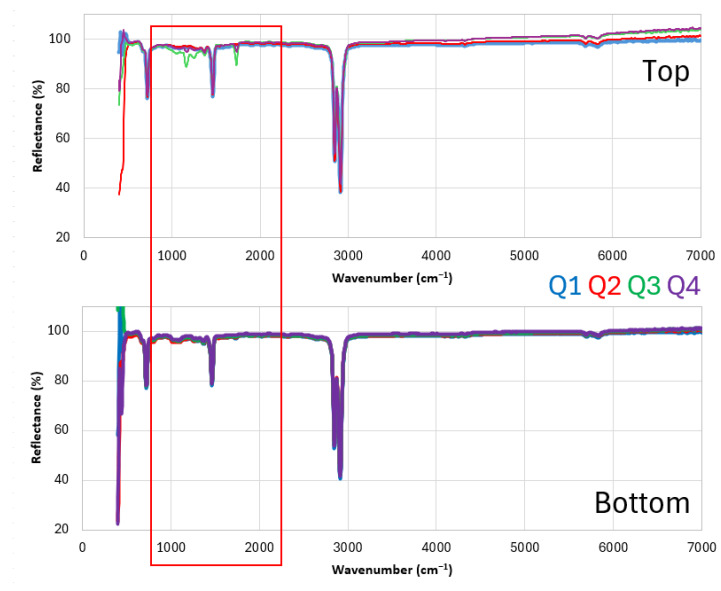
FTIR analysis of the top and bottom layer of the flight sample; red box underlines the area with new bands.

Figure 8 presents the FTIR spectra acquired from four regions of the flight sample, one for each sample quadrant, on both the top and bottom layers of the flight sample. The presented spectra summarize the most significant alterations observed in the sample due to exposure to the LEO environment. The spectra are essentially superimposable for the bottom layer, whereas significant alterations are observed in the top layer, which was directly exposed to atomic oxygen.

## 4. Discussion

The multilayer shield maintained its structural integrity throughout the MISSE-9 exposure. No measurable changes in thickness were observed, and mass variations remained within the experimental uncertainty associated with sample assembly and disassembly. Furthermore, no evidence of cracking, delamination, or erosion-related damage was detected. These results indicate that the compression-molded multilayer architecture is compatible with long-term exposure to the LEO environment. Consequently, the proposed experiment may open a new route in the field of manufacturing flexible cosmic-ray shielding materials. In previous MISSE campaigns, coated and filled polymers have been exposed; however, none featured a multilayer architecture. The concept of compression-molding thin films with embedded powders is also innovative, at least in the context of shielding applications.

In the future of human space exploration, an increasing variety of materials with new functionalities will be required. Suits and wearable devices must be flexible while providing additional protection in harsh environments. The compression-molded thin sheets (Figure 4), filled with BN and Sm–Co, showed excellent performance in the LEO environment, with no evidence of delamination or cracking.

Despite the prolonged exposure (>6 months), the LEO environment produced only limited physical degradation. Both the weight and the thickness of the individual layers are unaltered (Table 1). Small differences before and after exposure may depend on the disassembly procedure, as the original disks were measured before assembly, whereas joint separation at the end of the mission could have led to an irregular redistribution of the melted polymer between the disks. Moreover, a small thickness variation of about 5 µm for each disk partially affects the measurement reproducibility.

In terms of mass, M9W-C9 weighed 289.3 mg before exposure and 290.8 mg after. The corresponding values were 300.4 mg and 297.9 mg for the control sample, thus confirming that the flight sample did not undergo significant mass changes due to exposure.

As expected, the atomic oxygen concentration in the wake orientation is minimal. On average, the full thickness of the shield (560 µm) remained unchanged throughout the entire campaign and was not influenced by the space environment.

Nevertheless, the flight sample can be visually distinguished due to its yellowing (Figure 6), whereas the surface of the control sample remains unchanged. Oxidation occurred during exposure, although its origin is not entirely clear. Typically, atomic oxygen can induce surface oxidation; however, its concentration in the wake orientation is extremely low, and erosion would also be expected if it were the dominant mechanism. The atomic oxygen fluence in the wake orientation is estimated to be less than 10% of that in the opposite ram direction. Instead, no evidence of material removal was observed on the sample surface.

Oxygen could have originated from other sources, such as outgassing; however, MISSE materials are typically outgassed before launch, and outgassing generally produces additional effects on surrounding samples that were not observed in this case.

The observed oxidation is most likely attributable to atomic oxygen exposure. The low atomic oxygen flux likely oxidized only a few nanometers of polyethylene, and that the resulting oxidized surface has partially reduced the effectiveness of the erosion mechanism.

In fact, Figure 7 shows that partial yellowing also occurs in the inner layers, including the BN-filled layer. In contrast, the bottom layer, which corresponds to the lowest LDPE sheet in Figure 3, remains almost completely unaltered. The oxidation of the inner layers may be explained by the fact that bonding was performed only at four peripheral points of the assembly, allowing the LEO environment to enter between the sheets.

As shown in Figure 5, the assembled flight sample was firmly constrained within the MISSE carrier by its periphery. For this reason, this region was not exposed to atomic oxygen and did not exhibit yellowing (Figure 6). This clamping zone consisted of a solid annulus approximately 2.4 mm wide. The oxidation observed in the inner layers is likely attributable to atomic oxygen diffusing between adjacent sheets, due to the low oxygen permeability of LDPE.

Figure 7 also shows that the filled layers did not present a uniform particle distribution over the entire area, as some unfilled regions are visible. This is due to the compression-molding process, which caused partial redistribution of the deposited particles during processing. These unfilled regions allow proper bonding between adjacent LDPE layers and improve the flexibility of the sheet.

**Figure 9:**
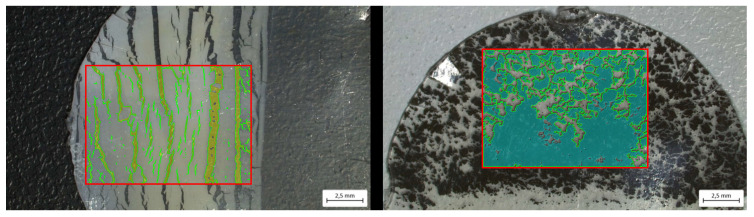
Details of the filling area evaluation for BN and SmCo layers.

In Figure 9, image analysis was used to calculate the filling area, which was 80.4% for BN and 73.2% for Sm–Co. Considering that the process is not a simple coating but a particle-filled system, this coverage level is relatively high considering the manufacturing approach.

FTIR analysis confirmed oxidation in the top layer, whereas no evidence of oxidation was detected in the bottom layer, independently of angular position on the disks, expressed in quadrants from Q1 to Q4. In general, the results from the different sheets are very similar, with minimal alterations observed in the internal layers and the bottom layer.

In Figure 8, a comparison of the full FTIR spectra of the external LDPE layers (top and bottom) is presented. The spectra of the bottom layer, acquired at four points corresponding to the different quadrants, are perfectly superimposed, as observed for virgin polyethylene and the backup sample. In contrast, the spectra of the top layer are not superimposed in the low-wavenumber range.

**Figure 10:**
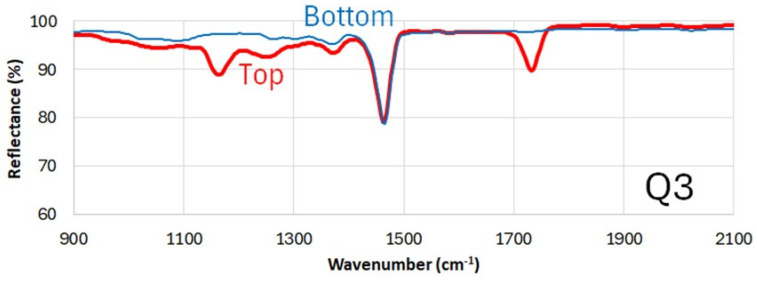
Detail of the FTIR spectra of the top and bottom layer of the flight sample.

A more detailed comparison is shown in Figure 10 for the third quadrant of the samples, where the highest alterations were observed in the top layer. The FTIR peaks of the top layer in the 1710–1740 cm^−1^ range correspond to C=O (carbonyl) groups, whereas the peaks in the 1100–1300 cm^−1^ range correspond to C–O groups. The different distribution of these peaks across the various regions of the top layer indicates that oxidation was not uniform.

## 5. Conclusions

The multilayer LDPE-based shielding architecture successfully completed long-term exposure aboard the MISSE-FF platform without exhibiting cracking, delamination, significant mass loss, or measurable thickness variations. FTIR analysis revealed oxidation primarily confined to the outermost layer, while the internal and bottom layers remained substantially unaffected. These results demonstrate the durability of the proposed multilayer design and its suitability for flexible shielding applications in the low Earth orbit (LEO) environment.

The study also demonstrated the feasibility of manufacturing multilayer shielding systems by combining compression-molded thermoplastic films with functional particle-filled layers. Although complete particle coverage was not achieved, the resulting architecture maintained good interlayer adhesion and structural integrity throughout the mission. Future process optimization will focus on increasing filler coverage while preserving adequate bonding between adjacent polymer layers.

At this stage, the investigation has been limited to minimally invasive characterization techniques, as the samples are part of an ongoing experimental campaign. Additional analyses, including high-resolution surface characterization and erosion assessment, will be performed after completion of the full testing program.

Overall, the results highlight the potential of multilayer LDPE-based systems for space shielding applications and demonstrate the capability of the MISSE-FF platform to support the in-orbit evaluation of innovative multifunctional materials. The proposed architecture also offers the possibility of simultaneously testing different shielding configurations within a single specimen, providing a versatile approach for future material development and qualification campaigns.

## Figures and Tables

**Table 1:** Physical data of flight and backup sample, before and after the MISSE9 exposure.

	Before Exposure		After Exposure	
	Weight, mg	Thickness, µm	Weight, mg	Thickness, µm
**Flight**				
LDPE	49.3	107	49.3	102
SmCo-LDPE	102.4	159	102.8	160
BN-LDPE	89.1	184	89.4	196
LDPE	49.4	108	49.3	102
**Backup**				
LDPE	47.2	101	47.2	97
SmCo-LDPE	122.7	174	120.5	181
BN-LDPE	82.9	171	82.7	175
LDPE	47.6	108	47.5	102

## Data Availability

The original contributions presented in this study are included in the article. Further inquiries can be directed to the corresponding author.

## Abbreviations

The following abbreviations are used in this manuscript:

BN	Boron nitride
GCR	Galactic cosmic ray
HDPE	High-density polyethylene
ISS	International space station
LDPE	Low-density polyethylene
LEO	Low Earth orbit
MISSE-FF	Materials International Space Station Experiment Flight Facility
PE	Polyethylene
Sm-Co	Samarium–cobalt
